# Genomic organization and evolution of the *ULBP *genes in cattle

**DOI:** 10.1186/1471-2164-7-227

**Published:** 2006-09-05

**Authors:** Joshua H Larson, Brandy M Marron, Jonathan E Beever, Bruce A Roe, Harris A Lewin

**Affiliations:** 1 Laboratory of Immunogenetics, Department of Animal Sciences, University of Illinois at Urbana-Champaign, 210 Edward R. Madigan Laboratory, 1201 W. Gregory Dr., Urbana, IL 61801, USA; 2 Laboratory of Molecular Genetics, Department of Animal Sciences, University of Illinois at Urbana-Champaign, 220 Edward R. Madigan Laboratory, 1201 W. Gregory Dr., Urbana, IL 61801, USA; 3 Department of Chemistry and Biochemistry, Advanced Center for Genome Technology, University of Oklahoma, 2107 Stephenson Research and Technology Center, 101 David L. Boren Blvd, Norman, OK 73069, USA

## Abstract

**Background:**

The cattle *UL16-binding protein 1 *(*ULBP1*) and *ULBP2 *genes encode members of the MHC Class I superfamily that have homology to the human *ULBP *genes. Human ULBP1 and ULBP2 interact with the NKG2D receptor to activate effector cells in the immune system. The human cytomegalovirus UL16 protein is known to disrupt the ULBP-NKG2D interaction, thereby subverting natural killer cell-mediated responses. Previous Southern blotting experiments identified evidence of increased *ULBP *copy number within the genomes of ruminant artiodactyls. On the basis of these observations we hypothesized that the cattle *ULBP*s evolved by duplication and sequence divergence to produce a sufficient number and diversity of ULBP molecules to deliver an immune activation signal in the presence of immunogenic peptides. Given the importance of the ULBPs in antiviral immunity in other species, our goal was to determine the copy number and genomic organization of the *ULBP *genes in the cattle genome.

**Results:**

Sequencing of cattle bacterial artificial chromosome genomic inserts resulted in the identification of 30 cattle *ULBP *loci existing in two gene clusters. Evidence of extensive segmental duplication and approximately 14 Kbp of novel repetitive sequences were identified within the major cluster. Ten ULBPs are predicted to be expressed at the cell surface. Substitution analysis revealed 11 outwardly directed residues in the predicted extracellular domains that show evidence of positive Darwinian selection. These positively selected residues have only one residue that overlaps with those proposed to interact with NKG2D, thus suggesting the interaction with molecules other than NKG2D.

**Conclusion:**

The *ULBP *loci in the cattle genome apparently arose by gene duplication and subsequent sequence divergence. Substitution analysis of the ULBP proteins provided convincing evidence for positive selection on extracellular residues that may interact with peptide ligands. These results support our hypothesis that the cattle ULBPs evolved under adaptive diversifying selection to avoid interaction with a UL16-like molecule whilst preserving the NKG2D binding site. The large number of ULBPs in cattle, their extensive diversification, and the high prevalence of bovine herpesvirus infections make this gene family a compelling target for studies of antiviral immunity.

## Background

The cattle *Major Histocompatibility Complex Class I-like Gene Family A *(*MHCLA*) was initially discovered in a cattle spleen cDNA library during a search for highly divergent mammalian genes [1]. Two transcripts, *MHCLA1 *and *MHCLA2*, were found to be members of the MHC Class I superfamily, encoding cell-surface transmembrane proteins containing α1- and α2-like domains, but no α3-like domain. These molecules have peptide sequence similarity to their homologues in other mammalian species, including the ULBP and RAET1 molecules in humans [2,3] and the H60, RAE1 and MULT1 molecules in mice [4-7]. To establish consistency with the human nomenclature, the cattle *MHCLA1 *and *MHCLA2 *genes are renamed *ULBP1 *and *ULBP2*, respectively, in this study. The function of cattle ULBP molecules is not known, but the human and mouse homologues have been demonstrated to interact with the NKG2D receptor, leading to activation of natural killer (NK) cells and T cell subsets in anti-tumour and infectious disease immunity [8]. In vitro studies have demonstrated that the soluble human cytomegalovirus (hCMV) protein UL16 interferes with the ability of ULBP1 and ULBP2 to interact with NKG2D, and co-expression of UL16 with ULBP1 or ULBP2 results in cytoplasmic retention of the ULBP molecules [2,9,10].

Southern blot analysis revealed the existence of a high copy number of *ULBP *genes in the cattle genome and seven other ruminant genomes. It was thus hypothesized that the cattle *ULBP *genes evolved rapidly by duplication and sequence divergence in response to selective pressure exerted by a viral pathogen(s). Extensive duplication of the cattle *ULBP *genes may serve to increase the repertoire of ULBP molecules able to bind NKG2D to initiate an immune response even in the presence of a UL16-like molecule [1].

The purpose of the present study was to identify the number of *ULBP *genes in cattle and describe their genomic organization. Six cattle bacterial artificial chromosome (BAC) clones were sequenced, resulting in the identification of 30 *ULBP *loci organized in two gene clusters on BTA9. Sequence analysis of the paralogues revealed that extensive gene duplication led to the present organization of the *ULBP *gene clusters. Bioinformatics tools were employed to characterize domains and sequence motifs in ten *ULBP *genes predicted to encode cell surface molecules, the majority of which are predicted glycoproteins. Substitution analysis identified specific codons in these genes that appear to be under positive Darwinian selection, and these selected sites were interpreted in a structural context using homology modelling.

## Results & discussion

### Identification of the minor and major *ULBP *gene clusters

Four minimally overlapping *ULBP*-containing BACs were identified by hybridization-based screening with a full-length cattle *ULBP1 *clone and then sequenced: RP42-147E22 [GenBank: AC092858], RP42-152A4 [GenBank: AC096629], RP42-146C17 [GenBank: AC098686] and RP42-194O5 [GenBank: AC098687]. Sequence alignment revealed that the former three BACs were overlapping, and the latter BAC was a singleton. Using BAC-end sequence data, two additional minimally overlapping BAC clones were identified: RP42-522F4 [GenBank: DQ405274] and CHORI240-21B24 [GenBank: DQ405273]. The overlapping clones were used to reconstruct two gene clusters, termed the "minor" *ULBP *cluster [GenBank: DP000082], spanning 331,973 bp, and the "major" *ULBP *cluster [GenBank: DP000081], spanning 464,586 bp (Table 1). The minor and major cluster sequences could not be further extended or joined by querying publicly available cattle genome sequence data [NCBI Build 2.0]. The *ULBP1 *locus [1] was not identified in this study, and therefore the major *ULBP *cluster sequence may be incomplete upstream of *ULBP7*.

Four *ULBP *loci were identified within the minor cluster, and 26 *ULBP *loci were identified in the major cluster. Nine loci represent coding sequences, and 21 loci are probable pseudogenes. Exons were identified by alignment and manual inspection (Table 2, 3). Loci were designated as genes if they contained uninterrupted coding sequence in the signal peptide, α1 and α2 domains. Loci either lacking an exon corresponding to the signal peptide, α1 or α2 domains or containing a stop codon in the coding sequence of one of these three domains were designated as pseudogenes. Many of the pseudogenes contain exons with intact coding sequence (Table 2, 3). It may be speculated that these pseudogenes serve as a repository for generating novel *ULBP *paralogues through gene conversion.

The nine *ULBP *genes identified in this study have a canonical five exon structure. An exception is *ULBP21*, which has six exons; the first two exons encode the signal peptide. All nine *ULBP *genes contain GU/AG exon splicing motifs. Because of the high degree of interlocus sequence identity among *ULBP *genes (e.g., *ULBP9 *and *ULBP27 *have 99.8% nucleotide identity over 1252 bp), the assignment of expressed sequence tags (ESTs) to any particular locus was problematic. Thus, EST data could not be used to definitively support *ULBP *gene annotation.

### Comparative genome organization

**Figure 1:**
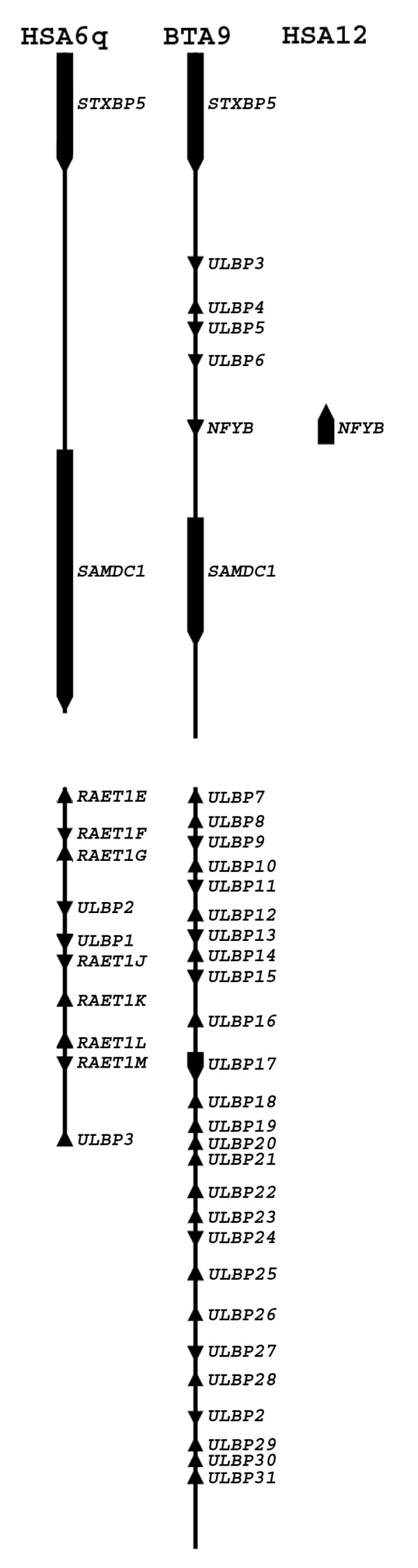
**Scaled comparative map showing homologous *ULBP*-containing chromosome regions in cattle and human. **Arrows indicate gene orientation. The BTA9 upper and lower seqments represent the major and minor *ULBP *clusters, respectively. Cattle BAC contig sizes and gene positions are found in Tables 1-3. The upper and lower chromosomal segments from HSA6q depict sequence positions 147,566–148,013 Kbp and 150,302–150,482 Kbp [NCBI Build 35], respectively. The HSA12 chromosomal segment represents positions 103,013–103,034 Kbp [NCBI Build 35].

Both the cattle minor and major *ULBP *clusters were localized to BTA9 using radiation hybrid mapping methods (data not shown). Comparative analysis showed that *STXBP5 *and *SAMDC1 *share a conserved orientation on HSA6q and BTA9 (Figure 1). The cattle *ULBP3, ULBP4, ULBP5 *and *ULBP6 *loci located between *STXBP5 *and *SAMDC1 *likely originated by duplication and insertion of genes from the major *ULBP *cluster (see below).

The cattle *NFYB *gene is orthologous to human *NFYB *on HSA12 (Figure 1). Comparison of nucleotide alignments in the cattle minor *ULBP *cluster and HSA12 genomic sequence demonstrates that sequence similarity is limited to the *NFYB *gene. The absence of genomic sequence similarity flanking this gene in humans and the lack of intronic sequence in cattle *NFYB *suggests that the cattle *NFYB *locus represents a retrotransposed gene. Although unlikely, a chimeric cattle BAC clone or sequence assembly error in the human genome cannot be ruled out as an explanation for these findings.

The discovery of at least 30 distinct cattle *ULBP *paralogues makes cattle the species with the largest number of *ULBP-*like genes identified to date (Figure 1). Our findings confirm and extend previous Southern blot analysis indicating a large number of *ULBP *paralogues in cattle and seven other ruminant artiodactyl genomes [1]. In contrast, the more distantly related artiodactyls, swine and alpaca, appear to have relatively few *ULBP *genes [1,11].

### The cattle *ULBP *loci evolved through extensive gene duplication

**Figure 2:**
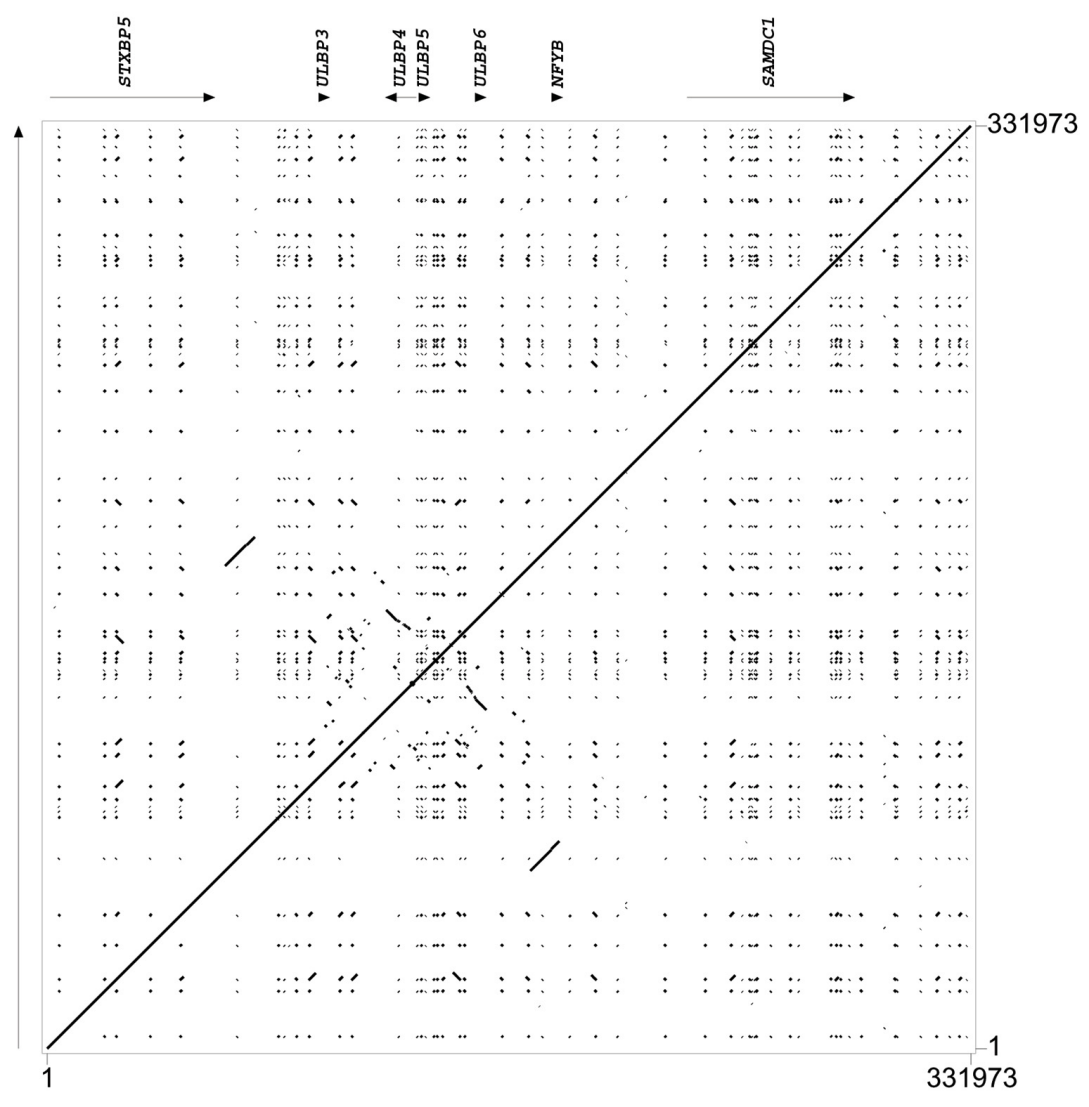
**Internal sequence identity plot of the minor *ULBP *cluster. **Sequence numbers displayed on the X- and Y-axes indicate alignment orientation, originating in the lower left corner. The central diagonal line represents identity; other lines indicate regions of internal sequence identity. Genes are annotated above the figure with arrows indicating orientation. Nucleotide sequences used to construct the minor *ULBP *cluster are listed in Table 1.

**Figure 3:**
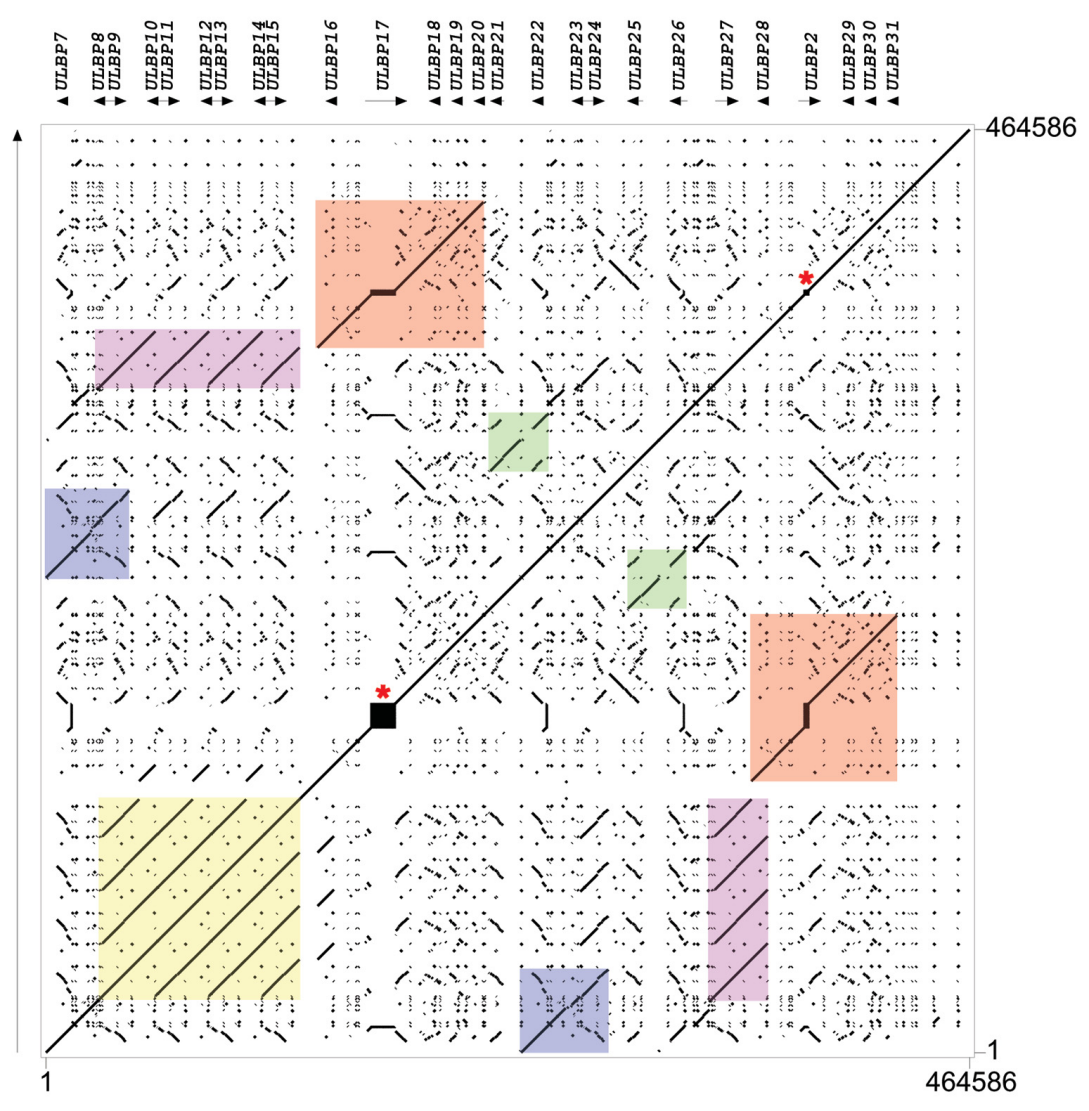
**Internal sequence identity plot of the major *ULBP *cluster. **Sequence numbers displayed on the X- and Y-axes indicate alignment orientation, originating in the lower left corner. The center line represents identity; other lines indicate regions of internal sequence identity. Genes are annotated above the figure with arrows indicating orientation. Nucleotide sequences used to construct the major *ULBP *cluster are listed in Table 1. The duplication of *ULBP28*-*ULBP31 *to form *ULBP16*-*ULBP20 *is within the red shaded area. The cattle specific repeat regions within *ULBP17 *and *ULBP2 *are indicated by red asterisks. The duplication of *ULBP7*-*ULBP9 *corresponding to *ULBP22*-*ULBP24 *is within the blue shaded area. The duplication of *ULBP21*-*ULBP22 *corresponding to *ULBP25*-*ULBP26 *is within the green shaded area. The tandem duplications of *ULBP9 *and *ULBP10*, *ULBP11 *and *ULBP12*, *ULBP13 *and *ULBP14*, and *ULBP15 *are within the yellow shaded area, and lines showing their similarity to the *ULBP27 *region are within the violet shaded area.

The cattle minor and major *ULBP *clusters were analyzed for internal nucleotide sequence similarity (Figure 2 and Figure 3, respectively) in order to identify duplicated segments. The largest was a duplication of *ULBP28*, *ULBP2*, *ULBP29*, *ULBP30*, and *ULBP31 *to form *ULBP16*, *ULBP17*, *ULBP18*, *ULBP19*, and *ULBP20 *(Figure 3). The directionality of this duplication event was determined from the expansion of a novel cattle-specific repeat (see below) in the first intron of cattle *ULBP17 *as compared to the smaller corresponding repeat region in the first intron of *ULBP2*. There appear to be four tandem duplications involving blocks containing *ULBP9 *and *ULBP10*, *ULBP11 *and *ULBP12*, *ULBP13 *and *ULBP14*, and *ULBP15 *(Figure 3). However, similarity to *ULBP27 *was observed for *ULBP9*, *ULBP11*, *ULBP13*, and *ULBP15*, thus providing evidence that *ULBP27 *was likely also part of the large duplication involving *ULBP28 *through *ULBP31 *described above. In addition to the duplication events described above, there are two other segments that contain duplicated genes: i) *ULBP7*, *ULBP8*, and *ULBP9 *are related to *ULBP22*, *ULBP23*, and *ULBP24*, and ii) *ULBP21 *and *ULBP22 *are related to *ULBP25 *and *ULBP26 *(Figure 3).

Known repetitive elements were identified in the minor and major *ULBP *clusters (Table 4). An additional novel genomic repeat was identified within the first introns of *ULBP17 *and *ULBP2*. The novel repeat spans 11,938 bp in the first intron of *ULBP17 *and 2,100 bp in the first intron of *ULBP2 *(Figure 3) [GenBank: DP000081]. These repeats are specific to the cattle major *ULBP *cluster and are not found elsewhere in the cattle genome. The large size of the *ULBP17 *repeat region relative to the corresponding repeat region in *ULBP2 *suggests active repeat expansion. A full understanding of the means by which these repeats contributed to the evolution of the *ULBP *gene family awaits complete genomic sequencing of this region and sequencing of additional haplotypes.

### Structure and evolution of ULBP proteins

**Figure 4:**
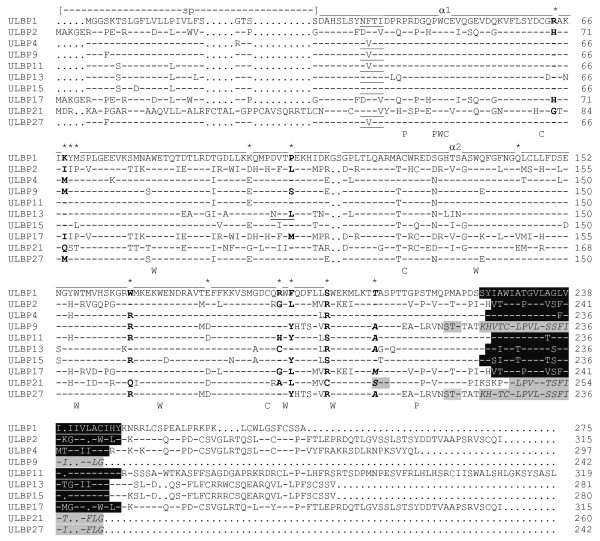
**Protein alignment of the cattle ULBP family. **Alignment gaps are represented by periods, and aligned ULBP sites sharing identity with cattle ULBP1 are represented by dashes. The signal peptide region (sp) is represented by dashes within brackets above the alignment. The α1 and α2 domains are designated by an underscore above the alignment. Universally conserved cysteine, proline, and tryptophan residues are annotated with Cs, Ps and Ws, respectively, beneath the alignment. Predicted N-glycosylation motifs are represented by underlined text in the alignment. Transmembrane domains are represented by black shaded background. Predicted GPI anchor motifs are represented by gray shaded background, and the associated downstream hydrophobic regions are indicated by italicized text with gray shaded background. Positively selected sites are indicated by asterisks above the alignment, and posterior probabilities associated with each positively selected site (see Table 2.5) are represented within the alignment by normal text (probability > 0.90), bold text (probability > 0.95), and italicized bold text (probability > 0.99).

**Figure 5:**
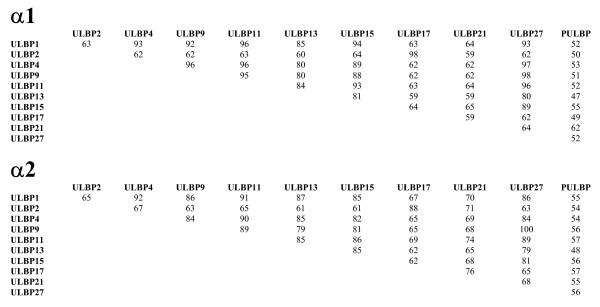
**Sequence identity in the extracellular domains ofcattle and swine ULBPs. **Percent pair-wise sequence identity between the α1 and α2 domains of ten cattle ULBP proteins and porcine ULBP [GenBank: AAP81932]. Sequence alignments were made using BLASTP and edited manually.

Conceptual translations of the nine cattle *ULBP *genes identified in this study and the previously identified cattle *ULBP1 *[1] are shown in Figure 4. Each molecule contains a 24 to 42 amino acid (aa) signal peptide sequence, an 88 aa α1 domain, an 84 aa α2 domain and a 25 to 30 aa connecting peptide region followed by a hydrophobic segment. Peptide sequence identity was determined within the α1 and α2 domains for each cattle ULBP and the porcine ULBP (Figure 5) [11]. ULBP9, ULBP21, and ULBP27 have glycophosphatidylinositol (GPI) anchor sites (P < 0.01, P < 0.05, and P < 0.001, respectively). The other seven ULBPs have predicted transmembrane domains of 23 to 25 aa followed by cytoplasmic tails ranging from 27 to 73 aa in length. The signal sequences and transmembrane domain or GPI anchor motifs indicate that all 10 of the expressed ULBPs are localized extracellularly. Each protein has predicted N-glycosylation motifs, with the exceptions of ULBP2 and ULBP17, suggesting that at least eight cattle ULBPs are glycoproteins.

**Figure 6:**
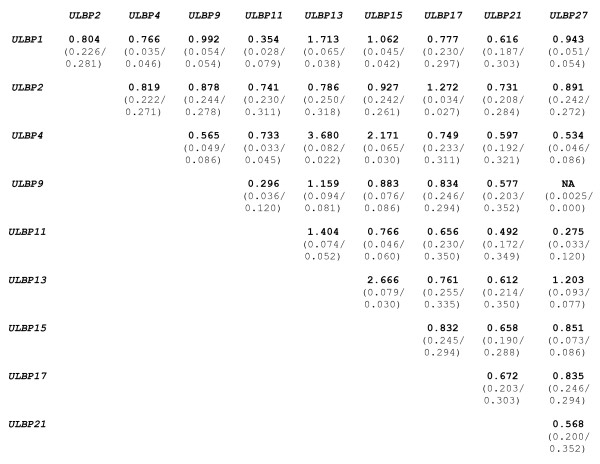
**Global substitution analysis of ten *ULBP *paralogues. **The bold numerical values correspond to ω_t _ratios; raw d_N_/d_S _values are listed in parentheses. Accession numbers associated with each sequence analyzed are listed in the Methods.

Pairwise substitution analyses of ten *ULBP *genes showed an average global nonsynonymous to synonymous substitution ratio (ω_t_) of 0.934 (Figure 6). Values of ω > 1.0 are regarded as indicating that positive selection has operated on the sequences analyzed [12]; however, global substitution analysis is stringent and may mask evidence of positive selection in molecular subregions [13]. Heterogeneity in selection intensity was investigated within the ULBP α1 and α2 domain regions (Table 5). In model 2 (M2), a continuous positive selection model with an additional (third) ratio of nonsynonymous to synonymous substitutions (ω_2_) estimated from the data, ω_2 _is 3.17, but represented only a small proportion (p_2 _= 0.08 out of 1.0) of codon sites. The log likelihood test of M2 vs. M1, the continuous neutrality model, was not statistically significant. In M3, the unconstrained discrete positive selection model, ω_2 _is 1.90 with p_2 _= 0.28. The log likelihood test of M3 vs M1 was significant, providing evidence of heterogeneity in ω ratios among codon sites. Model 8, a beta distribution with an added ω class estimated from the data, was compared to M7, a beta distribution that did not allow for positively selected sites. The log likelihood test of M8 vs M7 was significant, allowing the detection of positively selected codon sites (Table 5). Thirteen codon sites were determined to be under a high degree of positive selection (> 90% probability, Figure 4, Table 5).

**Figure 7:**
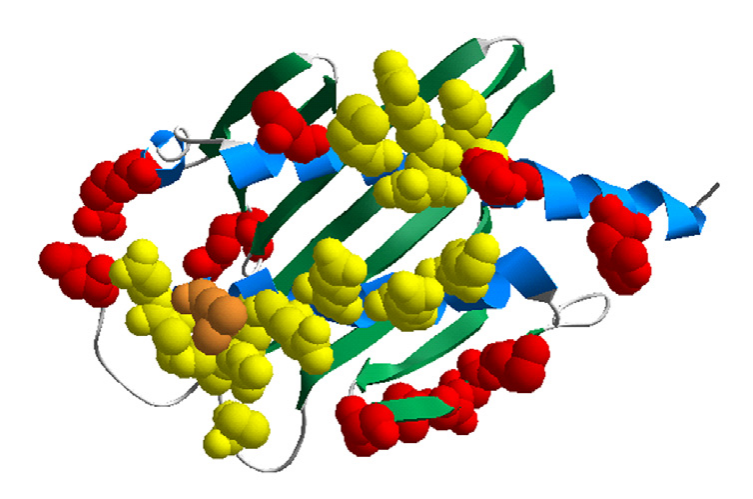
**Positively selected ULBP sites mapped onto the crystal structure of human ULBP3. **Positively selected cattle ULBP sites mapped onto the structure of human ULBP3. Tertiary structure of human ULBP3 [PDB: 1KCG, chain C] showing the spatial arrangement of homologous cattle ULBP residues under positive selection (>90% probability) as well as the human ULBP3 residues that interact with the NKG2D molecule. The human ULBP3 backbone appears in blue and green. Twelve of 13 cattle ULBP sites under positive selection are mapped onto the structure, and eleven appear as red space-filling residues. Thirteen of the fourteen human ULBP3 sites that interact with NKG2D appear as yellow space-filling residues. One site corresponding to both a selected cattle ULBP site and a human ULBP3 site that interacts with NKG2D appears in orange.

Twelve of the positively selected sites in the cattle ULBPs were mapped onto the three dimensional structure of human ULBP3 [PDB: 1KCG, chain C] (Figure 7). Eleven of the twelve positively selected residues were located at outwardly directed positions, indicating that positive selection acted at the level of interaction between the ULBPs and another molecule. On the basis of the structural data, fourteen human ULBP3 sites interact with NKG2D [14], and only one of these binding residues was found to overlap with the cattle ULBP sites under positive selection (Figure 7). Therefore, the positively selected cattle ULBP sites, located outside of the predicted NKG2D-binding residues on the basis of the homology modelling data, appear to interact with molecules other than NKG2D.

Several members of the Herpesviridae, which is the taxonomic family to which hCMV belongs, infect cattle, including bovine herpesviruses-1 through -5 and bovine lymphotrophic herpesvirus. The sequenced genomes of bovine herpesvirus-1, -4, and -5 do not encode molecules with detectable sequence similarity to UL16 of hCMV (HHV5); however it is conceivable that peptides encoded by the bovine herpesviruses or other viral pathogens may disrupt ULBP cell surface expression or the molecular interactions mediated by ULBP molecules. Thus, the rapid expansion of the *ULBP *gene family and the maintenance of such a large gene cluster are likely adaptive, serving to provide cattle with at least ten ULBP molecules through which an immune activation signal can be transmitted, even in the presence of an inhibitory pathogen-derived peptide.

## Conclusion

This study provides insights into the genomic organization and evolution of the cattle *ULBP *genes, a recently expanded MHC class I-like gene family in cattle with a probable role in antiviral immunity. For the first time, evidence of positive Darwinian selection on non-NKGD2-binding residues was obtained, strongly implicating immunogenic peptides as the driving force of molecular evolution of the cattle ULBPs. The stage is now set for studying the role ULBPs play in cattle immunity during infection by viral pathogens, as well as their organization and evolution in other mammals.

## Methods

### BAC selection, isolation and sequencing

To identify BAC clones containing *ULBP *genes, filter membranes containing the RPCI-42 male Holstein BAC library (12X genome coverage; Children's Hospital Oakland Research Institute) were screened by Southern blot hybridization using the full-length *ULBP1 *[GenBank: AF317556] cDNA clone as a probe. Probe amplification and labelling, membrane hybridization, washing conditions and autoradiography were performed as previously described [1]. *ULBP*-containing BACs were cultured in 3 ml 2x LB media with 20 μg/ml chloramphenicol (Sigma) overnight at 37°C with shaking. Cultures were centrifuged at 3000 × *g *for 3 min. The cell pellet was resuspended in 400 μl of a solution containing 0.05 M Tris, 0.01 M EDTA (pH 7.5) and 50 μg/ml RNase A (Sigma), lysed by addition of 400 μl of a solution containing 0.2 N NaOH and 1% SDS, neutralized by addition of 400 μl of a solution containing 4 M guanidine-HCl and 0.75 M KOAc (pH 4.6), and centrifuged at 10,000 × *g *for 10 min. An 860 μl aliquot of cleared lysate was combined with 600 μl isopropanol, placed on ice for 15 min, and centrifuged at 10,000 × *g *for 5 min. The supernatant was decanted, and the pellet was washed with 500 μl 70% ethanol before centrifugation at 10,000 × *g *for 5 min. The dried pellet was suspended in 40 μl of a solution containing 10 mM Tris and 1 mM EDTA.

The BAC DNA was digested using the *Hin*dIII restriction enzyme, separated by electrophoresis on 1X TAE agarose, stained using SYBR Green (Invitrogen), and visualized using image analysis (Typhoon Visual Imaging System, Molecular Dynamics) according to an established protocol [15]. Gel images showing restriction fragments were analyzed semiautomatically using IMAGE v3.10b [16], and band migration information was analyzed using FPC v6.0 [17,18] to determine clone overlap for contig assembly as previously described [15].

The first round of sequencing was performed for four minimally overlapping *ULBP*-containing BACs [GenBank: AC098687, AC092858, AC096629 and AC098686]. BAC DNA isolation, shotgun cloning, sequencing, quality analysis and sequence assembly were performed using established protocols [19,20]. The sequenced BACs were aligned using BLASTN v2.2.13 [21] to generate one contiguous genomic DNA sequence containing three BACs and one singleton. Repetitive elements in the contiguous sequences were masked using REPEATMASKER v3.1.3 [22] before the sequences were used to query publicly available cattle genome trace sequences [NCBI Build 2.0] to identify additional minimally overlapping cattle BACs. Two additional *ULBP*-containing BAC clones were identified [GenBank: DQ405273 and DQ405274], and a second round of sequencing was performed. Shotgun cloning and sequencing was performed using the Topo Shotgun Subcloning Kit (Invitrogen) according to the manufacturer's instructions. PHRED [23,24] was used to remove low quality sequence (PHRED score < 20). CROSSMATCH and PHRAP [25] were used to remove vector sequence and assemble BAC subclone sequences, respectively. For the two BACs sequenced in the second round, sequence gaps were closed by primer walking. Contiguous sequences [GenBank: DP000081 and DP000082] were constructed from overlapping full-clone BAC sequences using BLASTN. REPEATFINDER [26] was used to identify genomic sequence repeats not identified by REPEATMASKER.

### Gene annotation and bioinformatic analysis

Loci were identified in the repeat-masked contiguous genomic sequences by BLASTN alignment to the GenBank nonredundant [Release 151] and dbEST [Release 012006] databases and by BLASTX alignment to the GenBank nonredundant coding sequence database. The previously identified *ULBP1 *[GenBank: AF317556] and *ULBP2 *[GenBank: AY160681] sequences were aligned to the genomic sequences using BLASTN to assist in the annotation of the *ULBP *genes. Exon/intron boundaries were verified by manual inspection and editing. For each locus identified, all exons were joined and conceptually translated using SIXFRAME [27] to identify open reading frames. Homologous positions in the human genome [NCBI Build 35] were identified using the UCSC Genome Browser [28].

The ULBP multiple alignment was constructed using CLUSTALX [29]. Signal peptides and transmembrane domains were predicted using PSORTII [30], TMPred [31] and TMHMM v2.0 [32]. N-glycosylation and GPI-anchor predictions were carried out using NetNGlyc v1.0 [33] and big-PI predictor [34]. Homology modelling was performed using Swiss-Model and Swiss-PdbViewer [35]. Large-scale alignments were performed for the contiguous sequences using PIPMAKER [36].

### Substitution analysis

Ratios of nonsynonymous to synonymous substitutions (d_N_/d_S _or ω) were determined for the *ULBP *genes using the PAML software package [37] to identify evidence of positive Darwinian selection. Cattle sequences used for the substitution analyses included: *ULBP1 *[GenBank: AF317556], *ULBP4 *[annotated in GenBank: DP000082], *ULBP9*, *ULBP11, ULBP13*, *ULBP15*, *ULBP17*, *ULBP21*, *ULBP27 *and *ULBP2 *[annotated in GenBank: DP000081]. Only the extracellular α1 and α2 domain regions were analyzed. The YN00 program in PAML was used to estimate ω_t _for each group of aligned sequences using the method of Yang and Nielsen [38]. The CODEML program in PAML was used to identify variation in selection intensity. The data were modelled using maximum likelihood methods [39], and the results were compared to obtain a test statistic. Three comparisons were performed. Model M1, a neutrality model that constrained ω to be either 0 or 1, was compared to both M2, a selection model that added an additional ω ratio class estimated from the data, and M3, a selection model that used an unconstrained discrete distribution to model classes of ω ratios. This analysis used three discrete classes for M3. In addition, M7, a continuous distribution neutrality model that estimates ω using a beta function limited to the interval from 0 to 1, was compared to M8, a continuous distribution selection model that adds an additional class of sites with ω estimated from the data and not constrained to the interval between 0 and 1. A test statistic of twice the negative value of the difference between the log likelihood values generated under each model was compared to a χ^2 ^distribution with degrees of freedom calculated from the difference in the number of model parameters (M2 vs M1, df = 2; M3 vs M1, df = 4; M8 vs M7, df = 2). Posterior probabilities for *ULBP *sites under positive selection were generated under M8.

## Authors' contributions

JHL carried out sequence analysis and annotation, substitution analysis, homology modelling, interpretation of data, participated in the design of the study, and drafted the manuscript. BMM carried out BAC clone fingerprinting and selection of minimally overlapping clones. JEB performed sequence analysis and assembly and participated in the design of the study. BAR contributed BAC sequences. HAL supervised the research, participated in the design of the study, interpretation of data, and drafting of the manuscript. All authors have read the manuscript, provided critical reviews of content, and approved the final manuscript.

## Figures and Tables

**Table 1:** BAC clone composition of the assembled *ULBP *gene clusters

Assembled *ULBP *cluster (bp)	Component BAC clone (accession)	Size (bp)	Orient.	Component BAC sequence regions used in assembly	Corresponding *ULBP *cluster region
Minor *ULBP *cluster (331,973)					
	RPCI42-194O5 (AC098687)	156,543	+	5-156,543	1-156,539
	RPCI42-522F4 (DQ405274)	202,200	+	26,767–202,200	156,540–331,973
Major *ULBP *cluster (464,586)					
	CHORI240-21B24 (DQ405273)	116,254	+	1-98,955	1-98,955
	RPCI42-147E22 (AC092858)	165,590	-	165,584-28,446	98,956-236,094
	RPCI42-152A4 (AC096629)	191,732	-	185,338-121,525	236,095–299,908
	RPCI42-146C17 (AC098686)	164,686	+	9-164,686	299,909–464,586

**Table 2:** Genomic annotation of the minor *ULBP *gene cluster

Locus	Status	Orient.	Exon	Exon position (bp)	Size (bp)	Stop codon
*STXBP5*	gene	+	11	1,039–1,186	148	No
			12	1,390–1,456	67	No
			13	2,620–2,840	221	No
			14	3,330–3,508	179	No
			15	10,247–10,372	126	No
			16	12,700–12,851	152	No
			17	18,793–18,858	66	No
			18	43,553–43,645	93	No
			19	44,900–45,269	370	No
			20	45,574–45,739	166	No
			21	53,349–53,460	112	No
			22	57,390–57,614	225	No
			23	59,839–60,297	459	Yes
*ULBP3*	pseudogene	+	3	101,446–101,579	134	No
*ULBP4*	gene	-	1	132,643-132,512	132	No
			2	124,691-124,428	264	No
			3	124,189-123,914	276	No
			4	123,310-123,175	136	No
			5	121,996-121,521	476	Yes
*ULBP5*	pseudogene	+	2	137,458–137,605	148	No
*ULBP6*		+	2	154,996-155,258	263	Yes
			3	155,474–155,749	276	No
			4	156,396–156,527	132	Yes
			5	157,711–157,835	125	Yes
*NFYB*	gene	+	1	184,327–185,342	1,016	Yes
*SAMDC1*	gene	+	1	230,021–230,485	465	No
			2	290,210–290,272	63	Yes

**Table 3:** Genomic annotation of the major *ULBP *gene cluster

Locus	Status	Orient.	Exon	Exon position (bp)	Size (bp)	Stop codon
*ULBP7*	pseudogene	-	2	8,262-8,018	245	Yes
			3	7,790-7,515	276	No
			4	6,890-6,759	132	No
			5	5,578-5,454	125	Yes
*ULBP8*	pseudogene	-	2	24,180-23,979	202	Yes
*ULBP9*	gene	+	1	28,719–28,813	95	No
			2	37,552–37,815	264	No
			3	38,054–38,329	276	No
			4	38,934–39,066	133	Yes
			5	39,881–40,391	511	Yes
*ULBP10*	pseudogene	-	2	51,618-51,357	262	No
			3	51,115-50,835	281	Yes
*ULBP11*	gene	+	1	55,298-55,409	112	No
			2	64,155-64,418	264	No
			3	64,656-64,931	276	No
			4	65,535-65,667	133	No
			5	66,814-67,324	511	Yes
*ULBP12*	pseudogene	-	2	78,583-78,389	195	No
			3	78,079-77,816	264	No
*ULBP13*	gene	+	1	82,210–82,345	136	No
			2	91,064–91,327	264	No
			3	91,564–91,839	276	No
			4	92,443–92,578	136	No
			5	93,722–94,227	506	Yes
*ULBP14*	pseudogene	-	2	105,503–105,240	264	No
			3	105,005-104,733	273	Yes
*ULBP15*	gene	+	1	109,168–109,278	111	No
			2	117,844–118,107	264	No
			3	118,345–118,620	276	No
			4	119,224–119,356	133	No
			5	120,500–121,003	504	Yes
*ULBP16*	pseudogene	-	2	141,435-141,160	276	No
			3	140,931-140,666	266	No
*ULBP17*	gene	+	1	160,588–160,721	134	No
			2	178,284–178,547	264	No
			3	178,782–179,057	276	No
			4	179,685–179,817	133	No
			5	181,195–181,591	397	Yes
*ULBP18*	pseudogene	-	2	194,201-194,003	199	No
			3	192,969-192,694	276	Yes
*ULBP19*	pseudogene	-	2	205,757-205,495	263	No
			3	205,259–204,985	275	Yes
			4	204,053-203,923	131	Yes
*ULBP20*	pseudogene	-	3	215,364-215,116	249	Yes
*ULBP21*	gene	-	1	230,458-230,371	88	No
			2	227,628-227,590	39	No
			3	226,923-226,660	264	No
			4	226,284-226,009	276	No
			5	224,903-224,771	133	Yes
			6	223,621-223,508	114	Yes
*ULBP22*	pseudogene	-	2	247,327-247,005	263	Yes
			3	246,855-246,580	276	No
			4	245,961-245,830	132	No
			5	244,644-244,532	113	No
*ULBP23*	pseudogene	-	2	264,695-264,431	264	Yes
*ULBP24*	pseudogene	+	1	270,030–270,163	134	No
			2	277,941–278,215	275	Yes
			3	278,442–278,717	276	No
			4	279,606–279,738	133	Yes
			5	280,568–281,054	487	Yes
*ULBP25*	pseudogene	-	1	300,310-300,223	88	No
			2	296,899-296,861	39	No
			3	296,194-295,931	264	No
			4	295,553-295,279	275	Yes
			5	294,125-294,015	111	Yes
			6	292,846-292,384	463	Yes
*ULBP26*	pseudogene	-	1	322,695-322,562	134	No
			2	316,336-316,098	239	Yes
			3	315,865-315,589	277	Yes
			4	314,950-314,815	136	No
			5	314,113-313,670	444	Yes
*ULBP27*	gene	+	1	336,705–336,840	134	No
			2	345,589–345,852	264	No
			3	346,091–346,366	276	No
			4	346,971–347,103	133	Yes
			5	347,918–348,404	487	Yes
*ULBP28*	pseudogene	-	2	359,653-359,390	264	No
			3	359,148-358,883	266	No
			4	358,026-357,887	140	Yes
*ULBP2*	gene	+	1	378,496–378,629	134	No
			2	386,342–386,605	264	No
			3	386,840–387,115	276	No
			4	387,763–387,895	133	No
			5	389,276–389,762	487	Yes
*ULBP29*	pseudogene	-	2	402,249-402,066	184	No
			3	401,018-400,743	276	Yes
*ULBP30*	pseudogene	-	2	413,811-413,548	264	No
			3	413,313-413,038	276	No
			4	412,112–411,980	133	Yes
*ULBP31*	pseudogene	-	3	423,435-423,164	272	Yes

**Table 4:** Repetitive element composition of the minor and major *ULBP *gene clusters

Repetitive element	Minor *ULBP *cluster	Major *ULBP *cluster
SINE	5,681 bp (10.1%)	37,368 bp (8.8%)
LINE	9,973 bp (17.7%)	69,177 bp (16.3%)
LTR	2,406 bp (4.3%)	18,161 bp (4.3%)
DNA (including MER1/2)	2,388 bp (4.2%)	14,749 bp (3.5%)
Small RNA	0 bp (0.0%)	219 bp (0.1%)
Satellites	0 bp (0.0%)	0 bp (0.0%)
Simple repeats	893 bp (1.6%)	2,446 bp (0.6%)
Low complexity	394 bp (0.7%)	1,614 bp (0.4%)

Total repetitive elements	21,735 bp (38.6%)	143,734 bp (34.0%)

Repetitive element statistics were generated only from genomic regions flanked by *ULBP *loci. These include 56,390 bp of the minor cluster and 423,435 bp of the major cluster.

**Table 5:** Likelihood ratio tests of ω variation and identification of molecular sites under positive selection in ten cattle ULBP proteins

-2(M2 vs M1)	-2(M3 vs M1)	-2(M8 vs M7)	Parameter estimates for M8	Positively selected sites
3.2 (P < 0.25)	10.6 (P < 0.05)	8.1 (P < 0.025)	p_1 _= 0.279, p_0 _= 0.721 ω = 1.904, β (60.6, 99.0)	**64**, **68**, 69, 70, 99, **106**, 144, **165**, 178,**190**, **192**, **198**, ***206***

Accession numbers associated with the sequences analyzed are listed in the Methods section. M1, M2, M3, M7 and M8 refer to maximum likelihood models of ω ratios, and -2(M2 vs M1), -2(M3 vs M1) and -2(M8 vs M7) indicate the negative of two times the log likelihood difference between the selection and neutral models compared. P values for the test statistics are shown in parentheses. For M8, p_1 _is the proportion of positively selected sites, *p*_0 _is the proportion of sites not under positive selection, ω is the d_N_/d_S _ratio for the selected sites, and β(p, q) describes the beta distribution function. Positively selected ULBP sites are presented according to their numbered positions in the ULBP1 preprotein sequence. Posterior probabilities for positively selected sites are represented in normal text (probability > 0.90), bold text (probability > 0.95), and italicized bold text (probability > 0.99).
